# Digitalizing a Brief Intervention to Reduce Intrusive Memories of Psychological Trauma for Health Care Staff Working During COVID-19: Exploratory Pilot Study With Nurses

**DOI:** 10.2196/27473

**Published:** 2021-05-26

**Authors:** Laura Singh, Marie Kanstrup, Katherine Depa, Ann-Charlotte Falk, Veronica Lindström, Oili Dahl, Katarina E Göransson, Ann Rudman, Emily A Holmes

**Affiliations:** 1 Department of Psychology Uppsala University Uppsala Sweden; 2 Swedish Collegium for Advanced Study Uppsala Sweden; 3 Division of Psychology Department of Clinical Neuroscience Karolinska Institutet Stockholm Sweden; 4 Functional Area Medical Psychology Karolinska University Hospital Stockholm Sweden; 5 Department for Health Promoting Science Sophiahemmet University Stockholm Sweden; 6 Division of Nursing Department of Neurobiology, Care Sciences and Society Karolinska Institutet Huddinge Sweden; 7 Samariten Ambulance Stockholm Sweden; 8 Emergency and Reparative Medicine Theme Karolinska University Hospital Stockholm Sweden; 9 Department of Medicine, Solna Karolinska Institutet Stockholm Sweden; 10 School of Education, Health and Social Studies Dalarna University Falun Sweden

**Keywords:** intrusive memories, psychological trauma, prevention, pilot trial, COVID-19, digital intervention, remote delivery, cognitive science, person-based approach, mixed methods, co-design, health care staff

## Abstract

**Background:**

The COVID-19 pandemic has accelerated the worldwide need for simple remotely delivered (digital) scalable interventions that can also be used preventatively to protect the mental health of health care staff exposed to psychologically traumatic events during their COVID-19–related work. We have developed a brief behavioral intervention that aims to reduce the number of intrusive memories of traumatic events but has only been delivered face-to-face so far. After digitalizing the intervention materials, the intervention was delivered digitally to target users (health care staff) for the first time. The adaption for staff’s working context in a hospital setting used a co-design approach.

**Objective:**

The aims of this mixed method exploratory pilot study with health care staff who experienced working in the pandemic were to pilot the intervention that we have digitalized (for remote delivery and with remote support) and adapted for this target population (health care staff working clinically during a pandemic) to explore its ability to reduce the number of intrusive memories of traumatic events and improve related symptoms (eg, posttraumatic stress) and participant’s perception of their functioning, and to explore the feasibility and acceptability of both the digitalized intervention and digitalized data collection.

**Methods:**

We worked closely with target users with lived experience of working clinically during the COVID-19 pandemic in a hospital context (registered nurses who experienced intrusive memories from traumatic events at work; N=3). We used a mixed method design and exploratory quantitative and qualitative analysis.

**Results:**

After completing the digitalized intervention once with remote researcher support (approximately 25 minutes) and a brief follow-up check-in, participants learned to use the intervention independently. All 3 participants reported zero intrusive memories during week 5 (primary outcome: 100% digital data capture). Prior to study inclusion, two or more intrusions in the week were reported preintervention (assessed retrospectively). There was a general pattern of symptom reduction and improvement in perceived functioning (eg, concentration) at follow-up. The digitalized intervention and data collection were perceived as feasible and rated as acceptable (eg, all 3 participants would recommend it to a colleague). Participants were positive toward the digital intervention as a useful tool that could readily be incorporated into work life and repeated in the face of ongoing or repeated trauma exposure.

**Conclusions:**

The intervention when delivered remotely and adapted for this population during the pandemic was well received by participants. Since it could be tailored around work and daily life and used preventatively, the intervention may hold promise for health care staff pending future evaluations of efficacy. Limitations include the small sample size, lack of daily intrusion frequency data in the week before the intervention, and lack of a control condition. Following this co-design process in adapting and improving intervention delivery and evaluation, the next step is to investigate the efficacy of the digitalized intervention in a randomized controlled trial.

## Introduction

The mental health of health care staff exposed to stressful and traumatic events during their work in the COVID-19 pandemic is a number one research priority internationally [1]. This work-related exposure to psychologically traumatic events may have serious effects for staff, such as symptoms related to posttraumatic stress [2]. Brief interventions that prevent the buildup or recurrence of such symptoms and can be tailored around working life and delivered remotely are urgently needed.

Here, we focus on a brief intervention to target one focal symptom that can arise after exposure to traumatic events—intrusive memories [3]. Intrusive memories are defined as recurrent distressing sensory-perceptual impressions of the traumatic event that intrude into the mind involuntarily [3], typically in the form of visual images [4]. They are a core clinical feature [5] of posttraumatic stress disorder (PTSD) [3] and constitute a promising target for novel interventions [6]. Intrusive memories can be distressing in their own right and can impair work functioning, for example, by disturbing concentration [7]. One concern for health care staff is how such mental health symptoms might affect their ability to deliver high quality patient care [2]. A barrier for staff is how to fit time for treatment into an already overly burdened schedule.

Exposure to psychologically traumatic events presents a problem for health care staff working in the pandemic and will continue to be a problem once the pandemic is over. Before the pandemic, experiencing different traumatic events (either as a direct threat to themselves or a witnessed threat to patients) could lead to mental health difficulties such as PTSD in health care staff [8] as shown in studies including rescue workers [9], obstetricians [10], critical care nurses [11], and emergency nurses [12,13]. During the COVID-19 pandemic, health care staff have experienced much higher levels of exposure to potentially traumatic events and already reported increased posttraumatic stress symptoms [2,14,15]. For example, 35% of health care workers exposed to COVID-19 in China reported moderate to severe PTSD symptoms 1 month after onset of the COVID-19 pandemic [16]; 26% of health care workers in Italy scored above the cutoff for PTSD [17], 40% of intensive care unit (ICU) staff in the United Kingdom reported clinically significant levels of PTSD symptoms [2], and 64% of nurses in Jordan experienced acute stress disorder during the pandemic [18]. PTSD symptoms can impair work performance: 27% of medical workers who reported PTSD symptoms said it interfered with their work functioning [19] and 20% considered changing their job [20]. In addition to staff turnover, PTSD symptoms have also been related to burnout in health care staff [21].

Interventions to support health care staff need to be suitable for delivery in their work context. This includes being suitable for ongoing exposure to stressful events in the line of work (ie, repeatable); preventative use to keep staff well and working (ie, prevent the buildup of symptoms); scalability, simplicity, and brevity; and remote delivery to reduce the risk of virus transmission during a pandemic. The development of new interventions requires adaptation and detailed feedback from people with lived experience [22]. In that light, we will report a pilot study with health care staff (N=3) working with patients with COVID-19.

Good evidence-based treatments for PTSD exist (eg, trauma-focused cognitive behavioral therapy and eye movement desensitization and reprocessing [23,24]). However, there is a lack of available therapists with the prerequisite training to deliver such treatments. Further, some health care staff perceive stigma related to mental health problems [20] and can be reluctant to undertake weekly psychotherapy given increased time demands in a pandemic. Little evidence exists for treatment effectiveness for people with ongoing trauma exposure, such as health care staff working in the pandemic [23,24]. Evidence-based treatment guidelines for PTSD [23,24] suggest that, when current treatments are lacking or ineffective, there may be utility in targeting single symptoms. We have proposed intrusive memories of trauma as a targeted single symptom [4,6]. For example, intrusive memories were reported by 65% of emergency nurses [12]. Drawing on cognitive science (eg, on mental imagery [25] and memory consolidation and reconsolidation), we have developed a behavioral intervention aimed to limit the occurrence or recurrence of intrusive memories.

The procedure takes approximately half an hour and is delivered according to a clear protocol and administered typically in one or two guided sessions (thereafter self-administered, if needed). Delivery currently requires guided support by someone trained in the intervention but does not always require a fully qualified mental health professional. The intervention consists of several components including a brief memory reminder to moments within the trauma (hotspots), training in mental rotation, and engaging in a visuospatial cognitive task (the computer game Tetris) for a specific time while actively using mental rotation (ie, planning ahead and visualizing in the mind’s eye how to rotate and move upcoming Tetris blocks to fit them into a horizontal line). The cognitive task aspect can be delivered on the participant’s own smartphone. This intervention does not require a detailed discussion of the traumatic event.

The rationale underlying the intervention includes the following. Engaging in the visuospatial task is hypothesized to compete for limited working memory resources [25] with mental imagery (sensory) aspects of the trauma memory. This in turn is hypothesized to limit the storage or restorage [26] of the sensory representations of trauma [27] and reduces the subsequent number of times that memory intrudes involuntarily.

The intervention can be used on the same day the traumatic event occurred (day 1 protocol) [28-30]. For intrusive memories from older traumatic events [31,32], there are seemingly minor but important procedural differences including the type of memory reminder instructions and the time between the memory reminder and the task (see Visser et al [26]). In protocols for older memories, participants are instructed that they will briefly bring to mind the visual image from a specific intrusive memory and then play the computer game Tetris using mental rotation for at least 20 minutes. For a more detailed discussion regarding the intervention, see Iyadurai et al [4] and Singh et al [6].

Early studies have shown that the intervention may prevent intrusive memory occurrence in patients soon after trauma [29,30] (eg, by 62% compared to attention placebo control in motor vehicle accident survivors [29]). In a study with patients with more diverse trauma types in a Swedish emergency department, participants in the intervention condition reported 48% fewer intrusive memories compared to attention placebo control at week 1 following the intervention and 90% fewer at week 5 [28]. Promising results in terms of established intrusion reductions have also been shown in small-scale case series research with refugees [32], patients with complex PTSD [31], and in a person with bipolar disorder and PTSD [33].

To date the intervention has been delivered in person. So that it can be delivered remotely, we have first taken steps to digitalize the instructions for this brief intervention [34]. Here, participants (clinicians, researchers, students) were generally positive toward the materials created, noting that they were clear, concise, and helpful. In addition, participants shared potential concerns about remote delivery, mainly regarding the need for real-time communication with target users [34].

As there is currently no gold standard for a systematic stepwise approach to use for developing a successful intervention, it has been recommended to collect and apply relevant steps suggested from different methodologies, to use them with a “flexible” approach [35], and to iteratively make adjustments based on stakeholders’ [36] and target users’ input [37]. Thus, the critical next steps in our intervention development following our initial work with digitalizing the intervention [34] involves getting feedback from target users. Therefore, we now piloted the intervention with digital study procedures and when adapted for the health care work context during a pandemic, and obtained target user feedback.

The aims of this mixed methods exploratory pilot study with health care staff with experience of working in the pandemic were to:

- Pilot the intervention procedures that we have digitalized (for remote delivery and with remote support) and adapted for this target population (health care staff working during a pandemic) to explore its ability to reduce the number of intrusive memories of trauma (primary outcome: week 5 diary post intervention) and improve related symptoms (eg, posttraumatic stress symptoms) and participant’s perception of their functioning (eg, concentration)
- Explore the feasibility and acceptability of both the digitalized intervention procedures and the digitalized data collection (eg, primary outcome measure)

## Methods

### Participants

Participants (N=3, all female) were Swedish registered nurses, all with a university education and in full-time employment, who had worked clinically in the ICU or ambulance service during the COVID-19 pandemic in the spring of 2020 and were still currently working. They were all around 50 years of age and had >30 years of experience in their work. They had specialized in anesthesia and intensive care or ambulance care. To protect participant anonymity, most demographic characteristics have been omitted.

The inclusion criteria were being 18 years or older; doing clinical work during the COVID-19 pandemic in hospital care facilities (eg, ICU, intermediate care, or ward), experiencing at least one traumatic event in relation to their clinical work as health care staff during the COVID-19 pandemic that met Diagnostic and Statistical Manual of Mental Disorders (Fifth Edition; DSM-5) [3] Criterion A for PTSD within the last 3 months, reporting memory of the trauma, experiencing at least two intrusive memories of work-related traumatic events during the COVID-19 pandemic during the week before inclusion, willing and able to briefly write these down, being fluent in Swedish, being alert and oriented, having access to a smartphone, having sufficient physical mobility to use their smartphone, willing and able to provide informed consent, and completing study procedures and willing to be contacted during the study. Exclusion criteria (consistent with our previous study with patients) were current intoxication or loss of consciousness >5 minutes.

### Measures and Materials

#### Participant and Traumatic Event Characteristics

Details of the traumatic events causing intrusive memories were assessed with a bespoke item (“Select which of the following category(s) best fits for the traumatic event(s) you have experienced during COVID-19 within the last three months and experience intrusive memories from”) and then 11 categories (including a category for *other* events) were presented. Event categories were based on existing literature on traumatic events in health care staff (eg, a traumatic or tragic death of a patient [13]). Experience of prior psychological trauma was assessed with the Life Events Checklist for DSM-5 [38] (see Table 1).

**Table 1:** Characteristics of the worked-related traumatic events that participants had intrusive memories from and prior experiences of traumatic events reported per participant.

Variable	Participant^a^	Participant	Participant
Time(s) of trauma	Between 1 and 3 months ago	Between 1 and 3 months; within the last 24 hours	Between 1 and 3 months ago; ongoing exposure
Traumatic event causing intrusive memories	Traumatic or tragic patient death; situation in which patient care did not work as planned	Traumatic or tragic patient death	Situation in which patient care did not work as planned; confronted with distressed family members of patients; *other* category: relative who did not dare to say farewell to a patient who was critically ill
Retrospectively rated number of intrusions in the week before study participation^b^	2	3	3
Prior experiences of traumatic event types (LEC-5^c,d^)	6	12	10

^a^Participant numbers are omitted to preserve anonymity.

^b^Assessed via a single item retrospective rating.

^c^LEC-5: Life Events Checklist for Diagnostic and Statistical Manual of Mental Disorders (5th Edition).

^d^A list of 17 traumatic event types. Here, we report the number of event types endorsed as “happened to me,” “witnessed it,” or “part of my job” [39].

#### Primary Outcome Measure

##### Number of Intrusive Memories of Trauma Post Intervention (Week 5)

The number of intrusive memories of the traumatic events was assessed with a digital adaptation of the pen-and-paper diary used in our previous work [28]. Instead of ticking a box for each intrusive memory during four time periods (morning, afternoon, evening, and night) on a paper diary, participants received four digital links per day (after each period of the day had passed) via SMS text message and email from the electronic platform SmartTrial [40] version 2020.1. Participants recorded their intrusive memories with this digital diary for 7 days starting 1 month after the intervention (ie, day 29 post intervention, week 5). In each link, they were asked how many intrusive memories they had during that time period (eg, in the morning) on a 9-point scale from *0* to *more than 7* (a follow-up question to specify the number appeared if they selected *more than 7*). The link also included a brief description of what intrusive memories are: “Intrusive memories are IMAGES from a traumatic event that pop suddenly into your mind, when you DO NOT WANT them to. (They are NOT the same as deliberately choosing to think about the event or thinking about it in words.) Please record EVERY intrusive memory you have had - even if it is the same one popping up several times. If you did not have any, please CHOOSE 0.” In addition to this brief description, participants had received more detailed instructions prior to commencing the diary (eg, an information video about the symptom *intrusive memories* and researcher support).

#### Secondary Outcome Measures (Including Intrusive Memory Measures)

##### Number of Intrusive Memories of Trauma Immediately Post Intervention (Week 1)

The number of intrusive memories of the traumatic events was also assessed in week 1 (starting after having received the intervention on day 1) in another identical 7-day digital diary.

##### Intrusion Questionnaire

The intrusion questionnaire (eg, [41]) was used to assess the frequency of intrusive or unwanted memories in the previous week (7-point scale from *never* to *many times a day*, with a follow-up question to specify the number if necessary) and the characteristics of the intrusive or unwanted memories (ie, distress, nowness, reliving, and disconnectedness) and whether different triggers are associated with the intrusive or unwanted memories of the traumatic events (101-point scale from 0 *not at all* to 100 *very strongly*). The retest reliability of the four scales assessing characteristics of intrusions ranges between 0.61 and 0.72 [42].

##### Impact of Event Scale-Revised: Posttrauma Intrusion and Avoidance Symptoms

The intrusion and avoidance subscales of the Impact of Event Scale-Revised (IES-R) [43] were used to assess the degree of subjective distress of posttrauma intrusion and avoidance symptoms. The IES-R shows high internal consistency (α=.96) and agreement with other measures of posttraumatic stress (eg, PTSD checklist: *r*=0.84) [44].

##### Posttraumatic Stress Disorder Checklist for DSM-5

Participants’ current symptoms of PTSD were assessed via the PTSD Checklist for DSM-5 (PCL-5) short version [45]. The PCL-5 short version accounts for 94.1% (*r*=0.97) of the variance in the original 20-item validated PCL-5 version [45] and has been specifically recommended for remote digital assessment after trauma [46].

##### Distress and Vividness of Intrusive Trauma Memories During Diary Weeks

Two self-rated items assessed participants’ level of distress and vividness associated with the intrusions (11-point scales from 0 *not at all* to 10 *extremely*). Ratings were collected within the diary at the end of week 1 and week 5.

##### Self-Rated Initial Intrusions (Baseline)

One item was used to assess how many intrusive memories the participant had experienced in the week prior to entering the study, from 2 to more than 7. If more than 7, a free-text response field to specify the number of intrusive memories was presented. This was followed by three self-rated items measuring the level of distress, vividness, or concentration disruption associated with the intrusions (11-point scales from 0 *not at all* to 10 *extremely*).

#### Other Prespecified Outcome Measures (Including Functioning Measures)

##### Self-Rated Concentration Disruption

Participants rated their perceived level of concentration disruption associated with intrusions with a bespoke item adapted from Holmes et al [7] (11-point scale from 0 *not at all* to 10 *extremely*).

##### Self-Rated Impact of Intrusive Memories on Functioning

A bespoke item was used to assess their perceived impact on daily functioning associated with the intrusions (“During the previous month how much did your intrusive memories of the traumatic event affect your functioning (social, occupational, or other important areas e.g. relationships with other people, work, parenting, schoolwork, housework, volunteer work etc.)?”) [4] (11-point scale from 0 *not at all* to 10 *extremely*).

##### Credibility/Expectancy Questionnaire of Doing the Intervention

Before the intervention, participants completed the Credibility/Expectancy Questionnaire [47], which included 5 ratings of treatment expectancy measuring to what degree the participant finds the intervention credible (wording adapted for this study).

##### Subjective Units of Distress

Subjective units of distress (SUD) were collected three times during the intervention process to measure participants’ level of distress (11-point scales from 0 *no distress* to 10 *worst possible distress*).

##### Coping

Participants perceived coping during the COVID-19 pandemic was assessed via two free-text response field questions (eg, are there any specific factors you think have made it more difficult or easier for you to handle the COVID-19 situation and its consequences?).

##### Adverse Events

Adverse events [48] were assessed via a free-text response field asking about the occurrence of any health problems since the last contact.

##### Feedback Questionnaire About Participation

A feedback questionnaire consisting of nine bespoke items assessed participant’s experience of study participation. Items included (eg, “How acceptable was it to do the task?”; 11-point scale from 0 *not at all* to 10 *extremely*) and questions about what has happened since the study with a yes or no response (eg, “Have you had any psychological or medical treatment since you did the task?”) and items with a free-text response field (eg, “Do you have any other comments?”).

### Procedure

#### Recruitment and Instructions for Study Procedure

Participants were all recruited from the professional network of the research team (author AR). Data collection occurred from July 8 to August 28, 2020. Participants received study information materials via email and postally, and provided their written and informed consent prior to study procedures. They were contacted by the researcher (author MK) to set up a time for a remote digital meeting using Zoom [49] (Zoom Communications Inc; premium university account). In the session, which was scheduled around their work, participants received instructions on how to complete measures and the intervention via an online platform, and help to complete the intervention as needed, and they were encouraged to give feedback on all aspects of study procedures.

**Figure 1:**
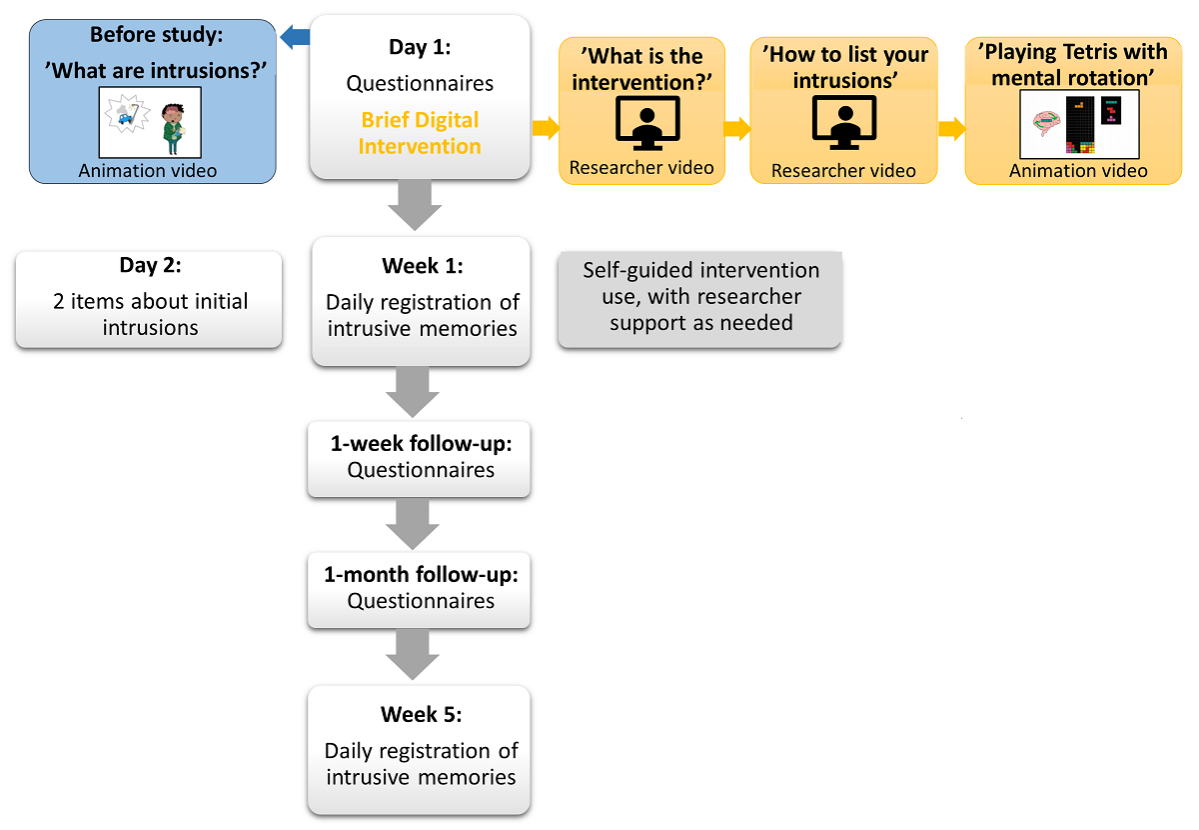
Flow of study procedures. Note that, in contrast to the planned follow-up randomized controlled trial, for these pilot participants, participation ended after the week 5 diary was complete.

The intervention was administered fully remotely and digitally. Remote researcher support during the procedure and delivery of the intervention was provided by a clinical psychologist with extensive experience in delivering the intervention to clinical participants (MK; see description of training in the Training to Deliver the Intervention section). During the remote meeting, participants received links to each step of the study (baseline measures; intervention package), and researchers (authors MK and KD) observed and took notes on feedback and questions that arose. The baseline and intervention session took approximately 1 hour in total (see Figure 1 for an overview of study procedures), depending on how much discussion arose. Of this, the intervention procedure itself took approximately 25 minutes. The overall approach here, with the intervention adapted for ongoing trauma exposure and self-use in a work context, was to deliver one intervention session with researcher support to teach participants how to do the intervention by targeting one selected intrusive memory and to promote continued use of the intervention to target any remaining or different intrusive memories and new intrusive memories should new traumatic events occur, with the option for researcher support in the initial week as needed.

#### Intervention Procedure

The intervention procedure commenced after the completion of baseline measures (see Figure 1). Participants watched a video (researcher video 1: 1:51 minutes) of a trained researcher giving an overview of the three parts of this intervention, that is, (1) a *brief memory reminder* so the chosen intrusive image is held in working memory before (2) engaging in a visuospatial interference task (playing the computer game *Tetris* via tetris.com) for *at least 20 min*, and (3) actively *using mental rotation* (ie, planning ahead and visualizing in their mind’s eye how to rotate and move upcoming Tetris blocks to fit them into a horizontal line). Next, they were instructed to briefly list their intrusive memories. They watched a video (researcher video 2: 2:03 minutes) on how to write this list and received written instructions in the platform. Instructions included “Please list your intrusive memories, using only a few words to describe what you ‘see’ when the intrusive memory pops up, e.g. ‘patient on ventilator’.” They were instructed to save this list (eg, screenshot) and then choose which memory to target in this session (eg, the most frequent or most distressing one). They were then instructed to “gently and briefly bring the chosen memory to mind” so they could *see it* in their mind’s eye (ie, for it to become active in working memory prior to the visuospatial interference task).

Next, a video and written instructions followed on how to play Tetris with mental rotation and how to access the game (via Tetris.com) and adjust the necessary settings (*ghost piece* off; animation video 3: 2:43 minutes). In this online version of Tetris, the game runs in *marathon mode*. Participants could choose to play on their smartphone or computer. Time between memory activation and game play was approximately 10 minutes (ie, the hypothesized time gap for memory to become malleable [26]). Prior to starting, they were reminded of their chosen memory and instructed to play for at least 20 minutes using mental rotation instructions and then to return to the platform.

#### Follow-up Procedures

After the intervention, participants were encouraged to keep using the intervention as needed and in a way that would fit with their work demands. They received instructions for completing an online daily diary registration on the number of intrusive memories over week 1 (see the Secondary Outcome Measures section). Incoming data was monitored approximately daily for the number of intrusive memories, and whenever intrusive memories were reported, participants had the option for *booster* doses, that is, they were reminded by the researcher (by SMS text message, email, or phone) to use the intervention themselves for the remaining intrusive memories and were given the option for researcher support (see the Results section).

At the 1-week and 1-month follow-ups, participants completed self-report questionnaires via the electronic platform. After the 1-month follow-up, they commenced with the primary outcome—daily registrations on the number of intrusive memories during week 5. Participants received an end-of-study letter that included a graph depicting their change in the number of intrusions over time. They were invited to a phone call with a researcher (MK) for additional feedback on study procedures to help refine these prior to starting the main randomized controlled trial (RCT).

### Training to Deliver the Intervention

The researcher delivering the intervention (MK) had received prior detailed training in delivering the intervention and how to obtain the primary outcome. This included theoretical and procedural knowledge; observing and being observed by a supervisor (author EAH) in role plays; receiving in-vivo and group supervision; and in this study, receiving regular ongoing supervision (from EAH) as necessary.

### Data Analysis

#### Quantitative Analysis

We conducted an exploratory analysis of quantitative results using a descriptive approach. Analyses and graphs were performed in Excel Professional Plus Version 16.0.5065.1000 (Microsoft Corporation).

#### Qualitative Analysis

A flexible approach building on content analysis as outlined by Bengtsson [50] was used. We conducted a qualitative analysis based on the notes that were taken by the researchers (authors MK and KD) during observing and discussing with participants in the digital intervention session with the researchers, from researcher–participant contact during the follow-up period, and open-ended questions in the electronic platform. The feedback was then organized based on emerging themes as an iterative process among the researchers.

Participant feedback was systematically evaluated to determine whether changes should be implemented, following the suggested person-based approach created by Yardley et al [51] and Bradbury et al [52]. This is a methodological approach for developing digital interventions (see also Gamble et al [34] for a detailed description of how this was carried out, including a description of how we applied the must have, should have, could have, won’t have [MoSCoW] method for prioritization [52,53]).

### Ethical Approval

This study was approved by the Swedish Ethical Review Board before the start of the study: 2020-03085. All participants provided their written and informed consent in accordance with the guidelines of the Declaration of Helsinki; signed consent forms were returned digitally and on paper. These pilot cases were done as a precursor to the planned RCT registered under ClinicalTrials.gov NCT04460014 (July 7, 2020).

## Results

### Characteristics of Traumatic Event Exposure and of the Intrusive Memories

Two participants experienced one recurring visual intrusive memory of a work-related traumatic event, and 1 experienced three separate recurring intrusive memories (ie, different visual scenes). Examples of these intrusive memories’ content included an image of patient’s faces, image of patient’s relatives, or image of a dead person (see Table 1 for additional information about the index traumatic events associated with these intrusions).

### Aim 1: Pilot the Digitalized and Adapted Intervention

All 3 participants completed all study procedures. In the past, we had used paper diaries, and here, the daily digital data capture of the number of intrusive memories via an electronic link (SMS text message or email) worked well (ie, the digital diary was completed by all 3 participants, and the feedback was positive). Our 3 participants answered 100% of the intrusive memory diary links, which were sent out four times a day for 7 days in a row, twice (primary outcome: week 5; secondary outcome: week 1 post intervention). The digital data capture for other questionnaires (secondary outcomes) was also successful (ie, questionnaires were completed by participants), with only two ratings missing for 1 participant (intrusion questionnaire: unwanted memory frequency and concentration disruption on day 2).

#### Primary Outcome

##### Number of Intrusive Memories of Trauma Post Intervention (Week 5)

**Figure 2:**
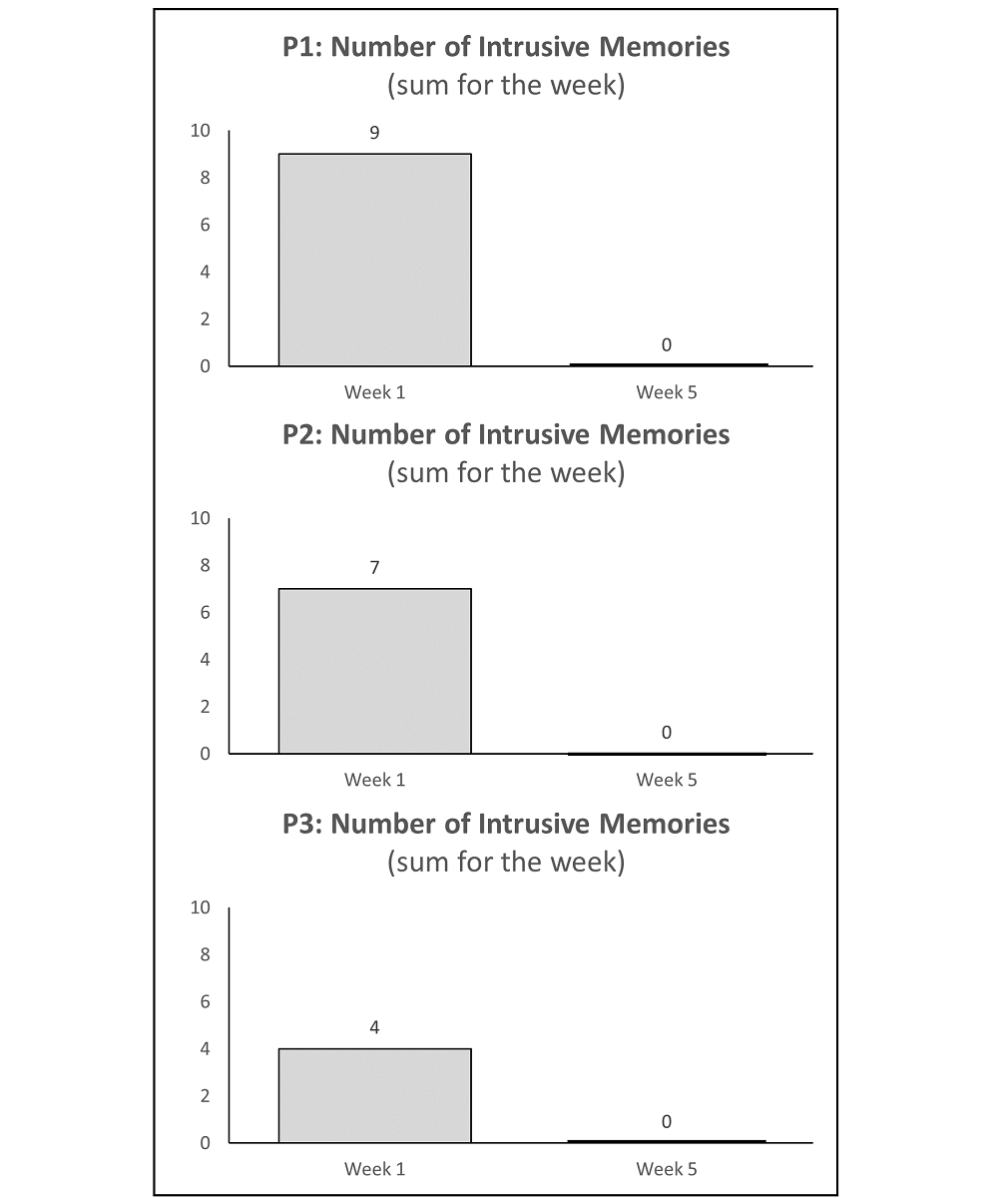
Graphs for visual inspection of the number of intrusive memories of trauma (sum for the week) during week 1 (secondary outcome) and week 5 (primary outcome) following the initial intervention session for each participant. After learning how to do the intervention (guided by the researcher) and practicing it independently during week 1, all 3 participants reported zero intrusions by week 5.

In contrast to baseline and week 1 postintervention levels (see the Secondary Outcomes: Number of Intrusive Memories [Week 1] section), all 3 participants reported 0 intrusive memories throughout the week 5 diary (Figure 2). Self-reported diary accuracy ratings were high (10, 6, and 10 out of 10). During discussion with the research team, the participant who rated a 6 explained that she was unsure if she should have also noted having a *normal* (ie, not intrusive) memory of the event when she no longer experienced intrusions.

#### Secondary Outcomes

**Figure 3:**
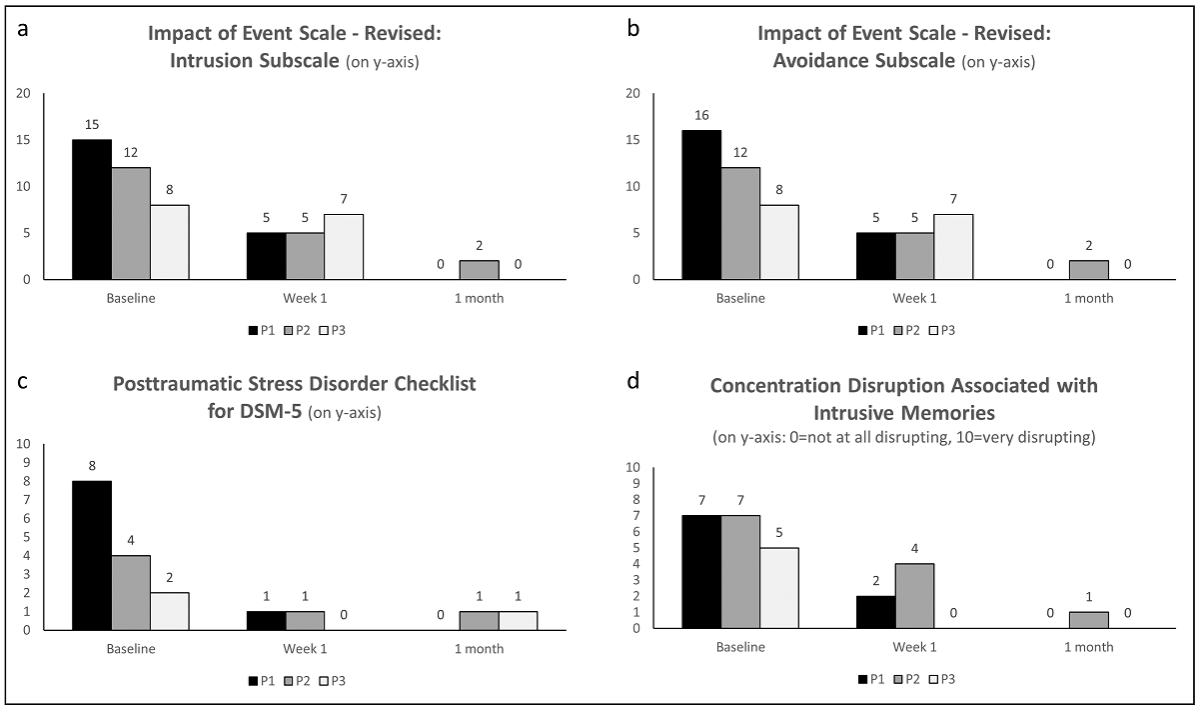
Bar graphs showing examples of intrusion-related symptoms (posttraumatic stress) and participant’s perception of their functioning in terms of concentration disruption (all secondary and other outcomes). All 3 participants reported reductions in intrusion (a) and avoidance (b) symptoms on the Impact of Event Scale-Revised from baseline to week 1 and the 1-month follow-up. Participant’s total scores on the Posttraumatic Stress Disorder Checklist for DSM-5 (c) decreased from baseline to week 1 and the 1-month follow-up. All 3 participants reported that their perceived concentration disruption associated with having an intrusive memory (d) decreased from baseline to week 1 and the 1-month follow-up. DSM-5: Diagnostic and Statistical Manual of Mental Disorders (5th Edition).

Multimedia Appendix 1 Table S1 shows secondary outcome data for each participant at all assessed time points, and Figure 3 displays bar graphs showing examples of secondary and other outcomes.

##### Number of Intrusive Memories of Trauma Immediately Post Intervention (Week 1)

In week 1, participants reported 9 (P1), 7 (P2), and 4 (P3) intrusive memories in total. Self-reported diary accuracy ratings were high for P1 and P3 (10/10), and medium for P2 (4/10).

We discuss lessons learned during the first week on a case-by-case basis. For all 3 participants, the intervention was delivered once with researcher support (approximately 25 minutes), and they subsequently used the intervention on their smartphone on their own. Participants received encouragement about intervention use from the researchers via 1 to 4 SMS text messages or emails (all participants) and one phone call (1 participant).

P1 reported 1 intrusive memory in the first half of the week and then a spike in intrusive memories on day 5 (7 intrusive memories). In communication with the researchers, she described that on this day she experienced an event at work similar to the one represented in her intrusive memory, which triggered these intrusions. She felt too tired to use the intervention straight after that work shift but successfully used it for that intrusion the day after.

P2 completed the game play part of the intervention on her computer on day 1. She reported 4 intrusive memories on the same day and 3 more on day 2. On day 2, she was instructed over the phone to repeat the intervention and encouraged by researchers to use her smartphone instead and to bring the image to mind before the game play. From day 3 to day 7 she reported no more intrusive memories.

P3 reported 2 intrusive memories on day 1 and 1 each on day 2 and day 3. From day 4 to day 7 she was intrusion-free, thus showing a general decrease of intrusion frequency over the week. She kept using the intervention during week 1 and wrote that she was “incredibly impressed by how such a simple thing could make such a huge difference.”

##### Intrusion Questionnaire

From baseline to week 1, the frequency of intrusive or unwanted memories of the traumatic event during the previous week slightly decreased from *twice a week* for 2 participants and remained the same (*twice a week*) for 1 participant. It then decreased to *never* after 1 month for all 3 participants (in line with the electronic diary data). The characteristics of the intrusive or unwanted memories—distress, nowness, and reliving—decreased from baseline to week 1 for all 3 participants. P1 and P2 reported a decrease in disconnectedness and in different triggers associated with the intrusive or unwanted memories from baseline to week 1, whereas P3 reported an increase from baseline to week 1. At 1 month, none of our 3 participants reported intrusions anymore, so characteristics were not rated.

##### Impact of Event Scale-Revised: Posttrauma Intrusion and Avoidance Symptoms

From baseline to 1 month, participants’ scores on the intrusion subscale decreased (from a range of 8-15 to a range of 0-2); similarly on the avoidance subscale, all scores decreased to 0 after 1 month (Figure 3a and b).

##### Posttraumatic Stress Disorder Checklist for DSM-5

From baseline to week 1, total scores on the PCL-5 decreased for all 3 participants and remained low at 1 month (range 0-1; Figure 3c). A similar pattern was shown in the intrusion subscale. P1 showed a decrease from 5 at baseline to 0 at 1 month. P2 and P3 showed a slight decrease from 2 at baseline to 1 at 1 month. Participants’ scores on the remaining subscales were already very low at baseline (0 or 1, with the exception of P1’s score of 3 on the avoidance subscale) and remained at or decreased to a very low level at 1 month.

##### Distress and Vividness of Intrusive Trauma Memories During Baseline and Diary Weeks

From baseline to week 1, vividness and distress associated with the intrusive memories decreased. P1 showed a strong decrease of vividness (7 to 3) and distress (7 to 2) from baseline to week 1, P2 showed a slight decrease from baseline to week 1 (vividness 10 to 9, distress 5 to 4), and P3 showed a strong decrease of vividness (10 to 2) and a slight decrease in distress (1 to 0) from baseline to week 1. At 1 month, none of our 3 participants reported intrusions anymore, thus related vividness or distress was not rated.

#### Other Prespecified Outcomes

Here we present outcomes regarding functioning and intrusive memories as used in our previous work [4,7,28]. We also present ratings of credibility and expectancy for the intervention, SUD experienced during the intervention procedure, coping, and adverse events (see Multimedia Appendix 1 Table S2 for details and remaining prespecified outcome data for each participant at all assessed time points).

##### Self-Rated Concentration Disruption

At baseline, concentration disruption associated with having an intrusive memory was medium to high (5 and 7 out of 10) and reported as lasting for 1 to 5 minutes per intrusive memory. At 1 month, concentration disruption had decreased to 0 or 1 out of 10 (Figure 3d). At week 5, none of our 3 participants reported intrusions anymore and thus did not rate associated concentration disruption.

##### Self-Rated Impact of Intrusive Memories on Functioning

From baseline to 1 month post intervention, 2 participants reported that the impact of intrusive memories on functioning decreased (from 5 [P1] or 2 [P3] out of 10 at baseline to 0 at 1 month), and it remained low for P2 (1 out of 10 at baseline and 1 month).

##### Credibility/Expectancy Questionnaire of Doing the Intervention

Credibility ratings taken after a brief description of the intervention prior to engaging in it were low to midrange for all 3 participants (38 out of 50 by P1, 35 by P2, and 21 by P3), and this was also reflected in qualitative feedback (eg, P3 wrote at the 1-month follow-up, “...[I] didn’t believe at all that this would work!”).

##### Subjective Units of Distress

During the intervention session with the researcher, participants completed distress ratings (SUD) before and after describing their intrusive memories, and after playing Tetris. For 2 participants, distress increased (P1 from 2 to 6; P3 from 0 to 1) but did not reach ceiling after describing and choosing an intrusion to target with the intervention, indicating successful emotional memory activation (note, P2’s ratings decreased from 2 to 0). Critically, after game play, distress decreased or remained at zero for all 3 participants (P1: 1; P2: 0; P3: 0).

##### Coping

In the end of the intervention session, we asked participants if any specific situations or factors made it difficult to cope with the COVID-19 situation at work and which made it easier to cope. They noted that, for example, support from the employer and help and support from colleagues made it easier to cope, and a lack of knowledge about COVID-19, lack of competence, difficulties in accessing and using personal protective equipment, and not being able to give person-centered care made it more difficult.

##### Adverse Events

There were no reported adverse events within the platform at week 1 and the 1-month follow-up.

### Aim 2: Feasibility and Acceptability of the Digitalized Intervention

#### Intervention

The digitalized intervention (approximately 25 minutes) was delivered with the option for researcher support (via video call). There was no dropout in our 3 participants. The session with the researcher and subsequent use of the intervention was flexibly scheduled into participant’s daily work and life. After this initial contact, limited researcher support was needed and none of the 3 participants requested additional support on how to use the intervention. Only 1 participant received a phone call on day 2; for the others, only brief encouragement to use the intervention via SMS text message or email was used.

#### Outcome Data Capture

All primary and secondary outcome data was successfully collected digitally or remotely. Digital procedures (eg, SMS text message) for reminding participants to fill in the daily registration on intrusions were used if needed.

#### Feedback Questionnaire About Participation

In a feedback questionnaire completed at 1 month, the intervention task was rated by participants 1 and 3 to be easy, and low in terms of how upsetting it was (see Table 2). One participant (P2) rated the task itself low in terms of easiness, noting later that the Tetris game play component was not easy. All 3 participants rated the intervention task to be acceptable. All 3 participants had used the intervention several times on their own after the initial session with the researcher. For instance, P1 reported that the work context triggered her intrusive memories and found it helpful to be able to use the intervention on her own in this setting. Participants had all mentioned the intervention to others, for example, to their friends or colleagues. None of the 3 participants reported receiving any other treatment since they took part in the study. Critically, all 3 indicated that they would highly recommend the intervention task to friends or colleagues.

**Table 2:** Responses on feedback questionnaire about participation from each participant at 1 month.

Feedback questionnaire about participation	P1	P2	P3
“If a friend or colleague has gone through a similar event, how likely is it that you would recommend this task?”^a^	10	10	10
“How easy did you find it to do the task?”^a,b^	9	1	10
“How upsetting did you find it to do the task?”^b^	1	0	0
“How much did you appreciate having something to do?”^b^	9	1	2
“How acceptable did you find the task?”^b^	9	10	10
“Received other treatment due to the traumatic event”^c^	No	No	No
“Did task on their own (i.e., after the researcher-led session)”^c^	Yes	Yes	Yes
“How often?”^c^	3-4	8	Daily for the first 2 weeks, after that every now and then
“Have you talked to others (e.g. friends, colleagues) about the task?”^d^	Yes	Yes	Yes

^a^Rated on an 11-point scale from 0 (not at all) to 10 (extremely/very much).

^b^In this study, *task* refers to the intervention as a whole. The wording in this questionnaire is adapted for use in the randomized controlled trial, where *task* can refer to either intervention or control.

^c^Free-text response field.

^d^Yes/no response.

#### Open-ended Feedback From Participants

In the open-ended feedback collected within the platform, the 3 participants expressed overall positive feedback about their outcomes after the intervention, that their experience with using the platform was overall good, and that they found the instructions and videos to be clear and helpful. P3 wrote as part of the open-ended feedback question given at the 1-month follow-up that the intervention was “excellent” in her case. P1 wrote in the platform both at the 1-week and 1-month follow-ups that she had not experienced any more intrusive memories since she had used the intervention on her own in the beginning of week 1. She noted that even when choosing to talk about the event of the memory with her colleagues she no longer experienced intrusive memories. Additionally, when contacted for a follow-up via phone call, participants reported that seeing their results gave them a sense of self-achievement (P1) and that they have learned a tool that they can use in future situations (P3).

In terms of impact on work functioning, P1 reported in the follow-up phone call that when she had intrusive memories it affected her empathy and her interactions with patients and relatives: “I did not dare to let them in, I was afraid something similar would happen and I would get more intrusive memories.”

##### Analysis of Themes in the Open-ended Feedback From Participants

We systematically analyzed participant feedback for changes or improvement, and three overarching themes emerged:

1. Getting the data right: This refers to minor changes in data collection (eg, clarifying the wording to some questions, removing items that were poorly understood, and adding a baseline diary; ensure quality of data).
2. Doing the digital intervention right: This refers to any changes in instructions, adding more information to further emphasize the important aspects, and for tech or intervention procedure (ensure intervention fidelity).
3. Feeling that study participation is alright: This refers to tailoring study procedures (eg, adding examples of staff categories in questions or examples) and adapting data collection as necessary (ensure participants feel included, can relate, and that participant burden is minimized).

##### Changes Made to the Study Materials Based on Feedback

Based on participant feedback, 18 changes were made (see Multimedia Appendix 1, Table S3 for a complete list). Eight were categorized under the theme “Getting the data right,” 7 under the theme “Doing the intervention right,” and 2 under “Feeling that participation is alright.” Based on the MoSCoW priority [52,53], 9 of the implemented changes were labeled as being a “Must Have,” such as adding “colleagues” as an example for the question on sources of social support, since health care professionals must abide by patient confidentiality rules, thus not being able to seek social support for work-related incidents in friends and family. Four points of feedback were labeled as being a “Should Have” and 6 as a “Could Have.”

Some changes that were suggested were not implemented, such as altering the original or translated wording of an official or pre-established questionnaire. Table 3 shows three selected examples of the implemented changes. For example, row 3 shows that P1 stated that it was difficult to recollect how many intrusions she experienced in the previous week. Based on this feedback, we proposed to our pilot participants as part of our follow-up phone call that future participants could complete a baseline diary that would begin at the time of enrollment. Pilot participants agreed that this would be a good addition to the study, and all reacted positively (eg, that it would provide a better baseline value of how many intrusions one experiences before compared to after the intervention), and they did not feel that adding an additional diary would be burdensome for participants. Thus, participants will only have to recall how many intrusions they have had within the last few hours and provide more accurate intrusion counts. This feedback was categorized as belonging to the theme “Getting the data right,” as this relates to participants’ being able to more accurately report the number of intrusions they have experienced.

Further, for the daily registration of intrusive memories, 1 participant said that it was easy to press the incorrect option indicating how many intrusions she had (eg, P1 accidently pressed “1” when she meant to press “0” for the week 5 diary). Overall, even though the digital intrusive memory registration was said to be a feasible and acceptable data collection method, there is a need for researchers to closely monitor and check in to verify the accuracy, as a participant can easily select the wrong option without being able to edit their response. Furthermore, we decided to take out the question about “diary accuracy,” as low responses given by some of the participants were due to confusion on what this really meant (ie, how precisely did they count their work-related intrusions or how well or how swiftly they entered their data into the platform after having received the link). 

**Table 3:** Examples of changes made to study materials and procedure following participant feedback [54].

Theme	Participant feedback as expressed by one or more participants	Changes made to the study based on feedback
Doing the digital intervention right	It is unclear what the next step is after filling out the baseline questionnaires, and after completing the assigned task. “*What happens now?*” (P1)	Added flowcharts that show the participant journey at the beginning and end of each module link that is sent out. It includes a green arrow with the text “You are here” to demonstrate where they are in the study and the next step.
Getting the data right	It is difficult to remember exactly how many intrusions one had in the last week (for the retrospective rating at baseline). (P1)	We kept this question as part of inclusion criteria and ask for a general estimate (ie, “have you had at least 2 intrusive memories in the last week”), but also added a baseline (week -1) daily electronic registration/diary of intrusive memories that participants are asked to complete during the week prior to filling in the baseline questionnaires and completing the intervention/control session.
Feeling that participation is alright	It looks like there are going to be a lot of questions in the SmartTrial list of questionnaires. [You] could lump some of the single-item, or shorter questionnaires together. (P3)	We did this with four questionnaires, where we combined two of them together twice. We also removed one of the work-related questionnaires.

## Discussion

### Summary of Findings

In this study with health care staff (3 registered nurses) who experienced intrusive memories in the context of the COVID-19 pandemic, we piloted a simple and brief intervention (here, approximately 25 minutes) with digitalized study procedures (for remote delivery and with remote support) that was adapted for this population (health care staff working during a pandemic). We explored whether the intervention reduced the number of intrusive memories of trauma (primary outcome) and improved related symptoms and participant’s perception of their functioning. All 3 participants reported a reduction in their intrusive memories (to zero) at week 5 post intervention, a greater reduction than expected; though, this has to be interpreted with caution given the small sample size. They also reported a reduction in other related symptoms—posttraumatic stress symptoms on the IES-R intrusion and avoidance subscales and the PCL-5. Of interest, all 3 participants perceived that their functioning improved (eg, they reported less disruption of their concentration). For example, P1 described that having intrusive memories affected her empathy and her interactions with patients and their relatives. This highlights that a reduction of intrusive memories might be beneficial for health care staff and for their patients and care.

We also explored the feasibility and acceptability of both the digitalized intervention and digitalized data collection. Participants perceived the intervention as feasible and rated it as acceptable (ie, they rated acceptability as 9, 10, and 10 out of 10). There was no dropouts (ie, all 3 participants completed the intervention), indicating favorable acceptability according to National Institute of Health and Care Excellence guidelines [23]; though, the small sample size is again noted. The primary outcome measure—a daily digital diary to capture intrusions—was successfully completed with zero missing data. Further, the secondary outcome measures (including another daily digital diary and digital questionnaires) were successfully collected remotely. Participants were overall positive about taking part in the study, the reduction in intrusion and other symptoms, and their perceived improvement in functioning (see the Open-ended Feedback From Participants section). They were also positive about the intervention as a useful tool that could be used in work and daily life. Our analysis of participant feedback led to changes in study materials and procedures in preparation for a subsequent RCT (see the Adjustments Prior to the RCT section).

Of particular interest is the notion that participants readily engaged in repeated self-use of the intervention to reduce remaining intrusive memories, those not targeted in the session with the researcher, or when their work triggered intrusive memories. This indicates high levels of user engagement with the intervention [55]. They described the intervention as a tool they could use in future situations and that they felt empowered by it. This is particularly important given the ongoing pandemic, which exposes health care staff working with patients with COVID-19 to potentially repeated traumatic events over sustained periods of time. The fact that this intervention is brief (only about 25 minutes) and can be used flexibly (eg, around shift work), on one’s own device, and does not require attending scheduled sessions with a mental health specialist is key for adoption given the high workload in this population. It also requires little input from the researchers (eg, one initial guided session, then little encouragement via SMS text message or email and only one phone call was needed).

To support health care staff during the pandemic and beyond, and to address broader critical challenges of reaching people affected by trauma at scale [56], interventions need to be not only simple and swift to deliver remotely but also repeatable. Clinical trials to test the effectiveness of this intervention are needed and should be adapted as necessary to different settings and trauma groups. If the intervention approach were proven effective, it may hold some useful features for implementation. For example, given that the intervention is brief and once learned can be self-administered, it could be used again (in effect as a booster session) following new trauma exposure. Thus, it may be particularly suitable for health care staff facing repeated or ongoing exposure to traumatic events in their lines of work and could be one of several possible measures to promote sustainability at work and well-being [57]. Psychological interventions to reduce trauma symptoms during ongoing exposure to trauma (eg, war or so called “frontline” work during a pandemic [1]) are currently lacking [24].

We note the number of intrusions reported at baseline was 2 to 3 per week, which is indicative of 8 to 12 per month. This may sound like a low number, but to the contrary, such levels can potentially cause significant distress and functional impairment; the gold standard assessment tool for PTSD (Clinical Administered PTSD Scale for DSM-5) states that an intrusion frequency of *only two intrusive memories per month* indicates a severity rating at the “moderate/threshold” level [58]. The intrusion reduction reported by our participants was to zero and perhaps reduced more than we might have expected given previous studies (eg, intrusion reduction in intervention condition vs control of 62% [29] and 48% [28,30] during week 1 and 90% during week 5 post intervention [28]). Therefore, we interpreted these results with caution given the small sample size and design used here. More generally, a symptom reduction of 50% may represent a clinically significant change, and such a drop in the number of intrusions might be a goal for this intervention approach rather than expecting total elimination.

In terms of perception of functioning, we note that participant’s self-rated concentration improved as their intrusions reduced. This is important as participants reported at baseline that their concentration was disrupted for approximately 1 to 5 minutes each time they experienced an intrusion. Why might having an intrusion, which disrupts concentration, at times lead to a problem of functioning at work? First, having even a brief but sudden and unplanned lapse in concentration after an intrusion can have the potential to interfere with the type of work-related duties that require focused attention, such as monitoring patients who are critically ill on a respirator. In our previous work, we have seen indication that intrusive memories can have a significant impact on people’s perception of their ability to concentrate [7,32]. Second, when the content of intrusive memories is of work-related traumatic events (eg, a difficult resuscitation, a typical situation reported by nurses as cause for intrusions [12]), the triggers for these intrusions are typically in work-related environments. This means that health care staff might be more likely to find work settings in which psychological trauma has occurred difficult, as those settings can trigger distressing memories, which could potentially lead to increased absentee rates due to avoidance of such work and reminders. The idea that having intrusions can lead to avoiding triggers of those intrusions is illustrated by one of our participants that related experiencing intrusions to their negative anticipations about going to work. Third, the content of reported intrusions can have a direct link to the ability to function on tasks at work that are similar to the intrusion content. That is, say the intrusive memory content is an image of a patient’s face and a tube, then this intrusive memory can be specifically triggered by other patient’s faces and tubes, rendering maximum disruption just at those specific situations at work where there is patient contact or fitting of a tube. By alleviating intrusive memories with specific content, the participant potentially regains or improves their ability to perform work tasks related to that content. For example, as described by our participants, the content of an intrusive memory (eg, including an image of a specific patient) from a traumatic situation involving a patient had a negative effect on their interactions with new patients (eg, perceived reduced capacity for providing empathic person-centered care). Thus, not having intrusive memories might also aid functioning at work. Were this to be the case, intrusion reduction techniques could be one among many strategies that will be needed to support staff and ultimately help reduce stress, burnout [2], and staff turnover [20].

To take steps that might help prevent burnout among health care staff is of high importance due to the fact that even before COVID-19 there was a global shortage of nurses [59]. The World Health Organization states that to address the nursing shortage by 2030, both educational efforts to increase the total number of nurse graduates together with an improved capacity to both employ and retain nurses in the health care system is necessary. Units such as high dependency departments are stressful environments and can lead to an even greater incidence of burnout [60], which might be further exacerbated during the COVID-19 pandemic [61,62]. Therefore, interventions that mitigate the impact of traumatic events are vital.

### Limitations

We noted several limitations of the current mixed methods exploratory pilot study. First and most important, since our study included a small sample (N=3), any changes in quantitative data reported by our 3 participants have to be interpreted with great caution.

Second, as a precursor to a planned RCT, we did not preregister this pilot study separately but did preregister the RCT before commencing this pilot (ClinicalTrials.gov NCT04460014, July 7, 2020), which reflects the outcomes reported here.

Third, we piloted only our intervention procedures in this study and can therefore only interpret reported intrusive memory data (primary outcome) compared to baseline assessment, not compared to a placebo control group. We will use an attention placebo control task, which we have piloted previously [28], in our planned RCT.

Fourth, we assessed the number of intrusive memories in the week prior to the intervention with a single time point retrospective rating. However, participants reported that it was difficult to remember how many intrusive memories they had during the previous week, posing a risk of the numbers we obtained through retrospective ratings being an under- or overestimation of the actual number of intrusions at baseline and making it difficult to compare numbers obtained through a retrospective rating at baseline with numbers obtained through a daily diary during week 1 and week 5. Following this limitation in this pilot study and because participants were positive toward completing an additional diary to monitor their intrusive memories at baseline, we will add a baseline diary in the planned RCT.

### Strengths

Strengths of this study include our use of different methodologies as part of stepwise intervention development [35] and including relevant organization stakeholders (end users with lived experience of health care staff working with patients with COVID-19 during the pandemic) as part of the development and implementation process [36]. Exploring the use of the digitalized intervention using a co-design approach together with the target user’s feedback has paved the way for optimizing intervention delivery for the next steps. Following Bird et al’s [63] checklist for feasibility of mental health interventions, we note that this first digitalized form of the intervention—once proven effective in a clinical trial—holds promise as a feasible intervention as it is cost saving, not time consuming, simple, applicable to the population of interest, matches prioritized goals, and no adverse events have hitherto been reported.

### Adjustments Prior to the RCT

Based on the target user feedback and lessons learned from this pilot study, we will make the following adjustments to the main RCT. In addition to adding a diary assessing the number of intrusive memories during the week before the intervention, we will also make changes to the diary itself. We will remove the accuracy rating at the end of the diary we had used in this pilot based on our previous pen-and-paper diary because it was unclear for our participants how to rate the item in relation to the digital diary. By collecting the diary data electronically (four times a day), we automatically obtain data on how compliant participants completed it (ie, how often they recorded their intrusive memories) in a more objective way compared to a subjective rating scale. Thus, we will use this data to report diary accuracy in the main RCT. Furthermore, being at work may trigger intrusive memories in this population of health care staff whose work also represents the context in which the traumatic events occurred. Therefore, we will add two ratings assessing the number of days at work and the number of night shifts in the previous week at the end of each diary. These ratings may help the researcher understand patterns of intrusions reported by participants (eg, high frequency of intrusions at night but none during the day could be because a person works night shifts).

We will also adapt the item assessing perceived functional impairment at baseline and follow-ups. Because participants found it difficult to answer our question on perceived functional impairment (since it included many different areas of functioning) and because our questionnaire measures of work-related stress did not seem to capture impairment at work due to intrusive memories very well, we will split this item into perceived work-related functional impairment (rating and an option for a free-text response on how work-related functioning was impaired because of having intrusive memories) and perceived functional impairment in other areas (eg, relationships with other people, social life, schoolwork, housework, or volunteer work). When asked in a phone call after week 5, participants were positive to this change. They also provided examples of perceived work-related functional impairment (see the Clinical Implications section).

We will also remove questionnaires on work functioning that did not reveal any effects in this pilot study to reduce participant burden. For example, a questionnaire assessing intention to leave the profession or workplace will be removed completely, and a questionnaire assessing burnout symptoms will only be assessed at baseline and the 6-month follow-up in the main RCT, since the effects of having intrusive memories on burnout might only unfold after longer time periods. Additionally, we will slightly change the wording of the intrusion questionnaire [41] from *unwanted memories* to *intrusive memories* to keep wording consistent across different measures in our study and because pilot participants did not understand the questionnaire as referring to intrusive memories (ie, the main focus of the study). Further, we will take out one irrelevant item (ie, a feedback question about appreciating having something to do, which we have used with emergency department patients but which does not make sense for this population), and we will use a modified version of the Credibility/Expectancy Questionnaire in the main RCT.

There was also some feedback from our participants we could not implement because of the limitations of the electronic data collection platform we are using in this study. For instance, we could not embed instruction videos directly in the electronic platform. Instead, participants had to click on a link directing them to a separate site on which we host the videos, which impaired study flow for participants. We also had to use a freely available version of *Tetris* (via tetris.com with the appropriate settings; see the Methods and Materials section), which included a 5 second commercial before being able to play, again impairing study flow. However, none of these issues were essential for intervention delivery or acceptability to our pilot participants. Finally, we learned from P2 that playing the game on the computer made it difficult to achieve flow. In the RCT, we will strongly encourage smartphone use for the game play task.

### Clinical Implications

As illustrated with participants P1 and P3, the recurrence of one specific intrusive memory (eg, recurrent images of a specific patient’s face) can directly impact related emotion and behavior (eg, having intrusions was reported as having an impact on the capacity to provide empathic patient-centered care or experiencing intrusions was related to negative anticipations about going to work). The perceived work-related impact from having intrusions described by P1 (see the Open-ended Feedback From Participants section) reflects a typical avoidance circle commonly seen in PTSD where similar stimuli as the trauma stimuli (here patients) triggered subtle avoidance behavior for P1. For P3, intrusive memories made her feel reluctant about going to work and worried that something similar would happen. P3 noted that prior to the intervention it felt as if her mental “backpack was totally full” and that experiencing intrusive memories warned her that she might experience more events at work. She was worried that encountering similar events would cause additional intrusions, that is, intrusive imagery in only *one* intrusive memory (from an event not considered particularly traumatic in itself, according to P3) brought on a sense of current threat and a strong negative emotional response [64,65]. P2 described that, after the intervention, the intrusive memories were just “normal memories.”

In this study, the intervention was delivered by a researcher who had been trained and had extensive experience on delivering the intervention with clinical participants (MK) and who received additional supervision as needed (from EAH). As training and supervision are currently deemed crucial to deliver the intervention adequately, we note that next steps in addition to the RCT include creating more standardized training material that can be delivered remotely (eg, in the form of an online training course) to researchers and procedures for role play, observation and feedback, and *in vivo* supervision.

Further, in this study, participants were recruited from within the professional network of the researchers. A potential barrier for recruiting a large sample for an RCT could be the heavy burden placed on health care staff during the pandemic, which might prevent them from engaging in a research study. Thus, recruitment strategies for an RCT need careful consideration and optimization.

### Conclusion

In this mixed method exploratory pilot study, we piloted a brief intervention that we have digitalized (for remote delivery and with remote support) and adapted for this target population (health care staff working during a pandemic). We explored whether the intervention reduced the number of intrusive memories of trauma (primary outcome) a month after the intervention session and improved related symptoms and participant’s perception of their functioning. Our participants (3 registered nurses who had experienced intrusive memories in the context of their work during the COVID-19 pandemic) reported zero intrusive memories at week 5 post intervention, a greater reduction than expected, which has to be interpreted with caution given the small sample size. Participants also reported a reduction in other related clinical symptoms (eg, posttraumatic stress symptoms) and perceived that their functioning improved (eg, they reported less concentration disruption).

We also explored the feasibility and acceptability of both the digitalized intervention and the digitalized data collection. After an initial session with the researcher, all 3 participants continued to use the intervention independently and perceived it as feasible and acceptable. The primary outcome measure (a daily digital intrusion diary) and secondary outcome measures (eg, digital questionnaires) were successfully completed. Should the intervention prove effective in future clinical trials, it could hold promise as a helpful tool, which because of its brevity and digital format, could be incorporated into health care staff’s work life and be repeated as needed in the face of numerous intrusive memories or ongoing trauma exposure. One next step will be to investigate the efficacy of the intervention in reducing the number of intrusive memories in an RCT with health care staff with exposure to traumatic events during the COVID-19 pandemic.

## Appendix

Multimedia Appendix 1 Supplementary materials.

## Abbreviations

DSM-5: Diagnostic and Statistical Manual of Mental Disorders (Fifth Edition)
ICU: intensive care unit
IES-R: Impact of Event Scale-Revised
MoSCoW: must have, should have, could have, won’t have
PCL-5: Posttraumatic Stress Disorder Checklist for Diagnostic and Statistical Manual of Mental Disorders (Fifth Edition)
PTSD: posttraumatic stress disorder
RCT: randomized controlled trial
SUD: subjective units of distress
