# Supplementary material for: Digitalizing a Brief Intervention to Reduce Intrusive Memories of Psychological Trauma for Health Care Staff Working During COVID-19: Exploratory Pilot Study With Nurses
Source: JMIR Form Res. 2021 May 26;5(5):e27473. doi: 10.2196/27473 (PMC8158532; doi:10.2196/27473)
Supplement: Multimedia Appendix 1 [file formative_v5i5e27473_app1.docx]

**Supplementary Material**

**Digitalising a Brief Intervention to Reduce Intrusive Memories of Psychological Trauma for Healthcare Staff Working During COVID-19: An Exploratory Pilot Study with Nurses**

Laura Singh^*^, PhD; Marie Kanstrup^*^, PhD; Katherine Depa, MA; Ann-Charlotte Falk, PhD; Veronica Lindström, PhD; Oili Dahl, PhD; Katarina E. Göransson, PhD; Ann Rudman, PhD; Emily A Holmes, PhD (* co-shared first authorship)

## **Supplementary Methods**

### **Measures and Materials**

***Other pre-specified outcome measures (including functioning measure)***

##### Self-rated social support

A bespoke item was used to assess perceived social support after the traumatic event (11-point scales from 0 = ‘none’ to 10 = ‘extreme/much’).

Negative appraisals of having intrusive memories

Negative appraisals of intrusive memories (two subscales: psychological problems and negative self-evaluations) were assessed via six self-report items taken from a previous appraisal measure (Newby & Moulds, 2010; 11-point scale from 0 to 100).

Self-rated sleep

Two self-rated items were used to measure the extent of being troubled by poor sleep (5-point scale from ‘not at all’ to ‘very much’) and the number of nights in the week with sleep problem (5-point scale from ‘0-1’ to ‘5-7 nights’). Scales are reverse scored. High scores indicate good outcomes.

Self-rated health

One self-rated item was used to measure perceived health status (Fýlkesnes & Førde, 1992; 7-point scale from ‘very good’ to ‘very bad’). Scales are reverse scored. High scores indicate good outcomes.

Time perspective questionnaire

The time perspective questionnaire measured participant’s time perspective via 8 self-report items assessing time perspective on three subscales: past perspective, present perspective and future perspective (Holman, Silver, Mogle, & Scott, 2016; 5-point scale from 1 = ‘not at all true’ to 5 = ‘very true’).

Questions related to work situation

Questions related to the work situation were assessed via three free text response field questions (e.g. which type of health care do you work with right now?) at baseline and two free text response field questions on whether the work situation changed and if yes, how, at follow-ups.

Sick leave

Sick leave was assessed via two bespoke items measuring the total number and the number of full workdays on sick leave because of reason for seeking health care.

Stress and Energy Questionnaire (SEQ): Stress symptoms and difficulty to let go of work related thoughts

The SEQ stress subscale was used to assess the frequency of feeling stressed, pressured and tensed at work during the previous week (5-point scale from 0= ‘never’ to 4 = ‘several times a day’; Kjellberg & Wadman, 2002). Further, a single bespoke item was used measuring difficulties to let go of work-related thoughts during leisure time (from 0 = ‘very rarely or never’ to 4 = ‘very often of always’) and a single question about whether the above-mentioned difficulties are because of intrusive memories.

Scale of Work Engagement and Burnout (SWEBO): Burnout symptoms

Participants’ symptoms of burnout was assessed using the burnout subscale from Scale of Work Engagement and Burnout (SWEBO) which consists of nine self-report items on three subscales: exhaustion, disengagement and inattentiveness (Hultell & Gustavsson, 2010; 4-point scale from 1 = ‘not at all’ to 4 = ‘all the time’).

Moral stress at work

Moral stress at work was assessed via five bespoke self-report items (4-point scale from 1 = ‘strongly agree’ to 4 = ‘strongly disagree’; developed based on Rudman, Omne-Pontén, Wallin, & Gustavsson, 2010). Higher scores indicate better outcomes.

Intention to leave job (ITL)

Intention to leave job was assessed via six self-report items on two scales: intention to leave profession and intention to leave workplace (4-point scale ranging from 1 = ‘strongly agree’ to 4 = ‘strongly disagree’, higher scores indicative of lower intention to leave profession/workplace). An additional item was added asking whether or not this was due to intrusive memories.

## **Supplementary Results**

**Table S1.** Secondary Outcome Measures for each Participant at Baseline, Day 2, Week 1 follow-up and diary, Week 5 diary, and 1 month follow-up

|  | **P1** | **P2** | **P3** |
| --- | --- | --- | --- |
| **Intrusion questionnaire: unwanted memory frequency** |  |  |  |
| Baseline | Once a day | Twice a week | Once a day |
| Day 2 | Missing data | Twice since study start | Twice since study start |
| Week 1 | Twice a week | Twice a week | Twice a week |
| 1 month^1^ | Never | Never | Never |
| **Intrusion questionnaire: unwanted memory characteristics** |  |  |  |
| **Distress** |  |  |  |
| Baseline | 70 | 38 | 9 |
| Week 1 | 30 | 22 | 0 |
| 1 month^x^ | N/A as zero intrusions | N/A as zero intrusions | N/A as zero intrusions |
| **Nowness** |  |  |  |
| Baseline | 68 | 50 | 18 |
| Week 1 | 1 | 31 | 0 |
| 1 month | N/A as zero intrusions | N/A as zero intrusions | N/A as zero intrusions |
| **Reliving** |  |  |  |
| Baseline | 70 | 51 | 40 |
| Week 1 | 30 | 4 | 6 |
| 1 month | N/A as zero intrusions | N/A as zero intrusions | N/A as zero intrusions |
| **Disconnectedness** |  |  |  |
| Baseline | 83 | 80 | 62 |
| Week 1 | 70 | 53 | 100 |
| 1 month | N/A as zero intrusions | N/A as zero intrusions | N/A as zero intrusions |
| **Triggers** |  |  |  |
| Baseline | 37 | 70 | 3 |
| Week 1 | 30 | 5 | 70 |
| 1 month | N/A as zero intrusions | N/A as zero intrusions | N/A as zero intrusions |
| **IES-R^2^** |  |  |  |
| **Intrusion subscale** |  |  |  |
| Baseline | 15 | 12 | 8 |
| Week 1 | 5 | 5 | 7 |
| 1 month | 0 | 2 | 0 |
| **Avoidance subscale** |  |  |  |
| Baseline | 16 | 2 | 3 |
| Week 1 | 4 | 1 | 2 |
| 1 month | 0 | 0 | 0 |
| **PCL-5**^3^ |  |  |  |
| **Total score** |  |  |  |
| Baseline | 8 | 4 | 2 |
| Week 1 | 1 | 1 | 0 |
| 1 month | 0 | 1 | 1 |
| **Intrusion subscale** |  |  |  |
| Baseline | 5 | 2 | 2 |
| Week 1 | 1 | 1 | 0 |
| 1 month | 0 | 1 | 1 |
| **Avoidance subscale** |  |  |  |
| Baseline | 3 | 0 | 0 |
| Week 1 | 0 | 0 | 0 |
| 1 month | 0 | 0 | 0 |
| **Negative alterations subscale** |  |  |  |
| Baseline | 0 | 1 | 0 |
| Week 1 | 0 | 0 | 0 |
| 1 month | 0 | 0 | 0 |
| **Arousal and reactivity subscale** |  |  |  |
| Baseline | 0 | 1 | 0 |
| Week 1 | 0 | 0 | 0 |
| 1 month | 0 | 0 | 0 |
| **Distress and vividness of intrusive trauma memories during baseline and diary weeks** |  |  |  |
| **Distress** |  |  |  |
| Baseline | 7 | 5 | 1 |
| Week 1 | 2 | 4 | 0 |
| Week 5 | N/A as zero intrusions | N/A as zero intrusions | N/A as zero intrusions |
| **Vividness** |  |  |  |
| Baseline | 7 | 10 | 10 |
| Week 1 | 3 | 9 | 2 |
| Week 5 | N/A as zero intrusions^4^ | N/A as zero intrusions | N/A as zero intrusions |

*Note.* ^1^Because of an error in the anchoring at 1 month follow-up, this questionnaire was assessed retrospectively during a phone call post-week 5. Participants responses to the erroneous questionnaire (assessing the frequency of unwanted memories since the beginning of the study) were ‘Several times a day’ (P1), ‘Twice a week’ (P2), and ‘Once a day’ (P3); ^2^IES-R, Impact of Event Scale Revised; ^3^PCL-5, Posttraumatic Stress Disorder Checklist for DSM-5; ^4^Ratings of vividness and distress associated with intrusive memories were not completed if participants had zero intrusions.

**Table S2.** Other Pre-specified Outcome Measures for each Participant at Baseline, Day 2, Week 1 follow-up and diary, Week 5 diary, and 1 month follow-up

|  | **P1** | **P2** | **P3** |
| --- | --- | --- | --- |
| **Concentration disruption (duration of disruption per intrusion if assessed)** |  |  |  |
| Baseline | 7 (1-5 min) | 7 (1-5 min) | 5 (1-5 min) |
| Day 2 | Missing data | 2 | 2 |
| Week 1 (diary) | 2 | 3 (4 in diary) | 0 |
| 1 month | 0 | 1 | 0 |
| Week 5 (diary) | N/A as zero intrusions^1^ | N/A as zero intrusions | N/A as zero intrusions |
| **Self-rated impact on functioning** |  |  |  |
| Baseline | 5 | 1 | 2 |
| Week 1 | 1 | 0 | 0 |
| 1 month | 0 | 1 | 0 |
| **Self-rated social support** |  |  |  |
| Baseline | 1 | 3 | 1 |
| Week 1 | 1 | 6 | 7 |
| 1 month | 1 | 1 | 8 |
| **Negative appraisals of having intrusive memories** |  |  |  |
| **Psychological problems subscale** |  |  |  |
| Baseline | 50 | 0 | 0 |
| Week 1 | 0 | 0 | 0 |
| 1 month | 0 | 0 | 0 |
| **Negative self-evaluations subscale** |  |  |  |
| Baseline | 0 | 0 | 0 |
| Week 1 | 0 | 0 | 0 |
| 1 month | 0 | 0 | 0 |
| **Self-rated sleep** |  |  |  |
| Baseline | 8 | 7 | 8 |
| Week 1 | 8 | 8 | 8 |
| 1 month | 8 | 7 | 8 |
| **Self-rated health** |  |  |  |
| Baseline | 6 | 7 | 7 |
| Week 1 | 7 | 7 | 7 |
| 1 month | 6 | 7 | 6 |
| **Time perspective questionnaire** |  |  |  |
| **Past perspective subscale** |  |  |  |
| Baseline | 6 | 7 | 3 |
| Week 1 | 4 | 10 | 4 |
| 1 month | 5 | 6 | 4 |
| **Present perspective subscale** |  |  |  |
| Baseline | 6 | 8 | 4 |
| Week 1 | 5 | 10 | 3 |
| 1 month | 5 | 10 | 3 |
| **Future perspective subscale** |  |  |  |
| Baseline | 12 | 9 | 14 |
| Week 1 | 12 | 14 | 12 |
| 1 month | 12 | 12 | 15 |
| **Questions related to work situation** |  |  |  |
| **Work situation changes** |  |  |  |
| Week 1 | No | No | No |
| 1 month | No | No | No |
| **Sick leave** |  |  |  |
| Baseline | 0 | 0 | 0 |
| Week 1 | 0 | 0 | 0 |
| 1 month | 0 | 0 | 0 |
| **SEQ**^2^ |  |  |  |
| **Stress subscale** |  |  |  |
| Baseline | 3 | 4 | 2 |
| Week 1 | 3 | 0 | 1 |
| 1 month | 3 | 0 | 0 |
| **Difficulty to let go of work-related thoughts during leisure time** |  |  |  |
| Baseline | 2 | 3 | 1 |
| Week 1 | 0 | 2 | 0 |
| 1 month | 1 | 1 | 0 |
| **Above mentioned difficulties because of Intrusive memories** |  |  |  |
| Baseline | Yes | No | No |
| Week 1 | No | No | No |
| 1 month | No | No | No |
| **SWEBO**^3^ |  |  |  |
| **Total score** |  |  |  |
| Baseline | 1.1 | 1.3 | 1.2 |
| Week 1 | 1 | 1 | 1.1 |
| 1 month | 1.2 | 1 | 1.2 |
| **Exhaustion subscale** |  |  |  |
| Baseline | 1.3 | 1.3 | 1 |
| Week 1 | 1 | 1 | 1 |
| 1 month | 1 | 1 | 1.3 |
| **Disengagement subscale** |  |  |  |
| Baseline | 1 | 1 | 1 |
| Week 1 | 1 | 1 | 1 |
| 1 month | 1.3 | 1 | 1 |
| **Inattentiveness subscale** |  |  |  |
| Baseline | 1 | 1.6 | 1.6 |
| Week 1 | 1 | 1 | 1.3 |
| 1 month | 1.3 | 1 | 1.3 |
| **Moral stress at work** |  |  |  |
| Baseline | 12 | 5 | 18 |
| Week 1 | 16 | 19 | 20 |
| 1 month | 12 | 17 | 20 |
| **ITL**^4^ |  |  |  |
| **Profession subscale** |  |  |  |
| Baseline | 15 | 15 | 15 |
| Week 1 | 15 | 15 | 15 |
| 1 month | 15 | 15 | 15 |
| **Workplace subscale** |  |  |  |
| Baseline | 15 | 14 | 15 |
| Week 1 | 15 | 15 | 15 |
| 1 month | 15 | 15 | 15 |
| **Above mentioned difficulties because of Intrusive memories** |  |  |  |
| Baseline | No | No | No |
| Week 1 | No | No | No |
| 1 month | No | No | No |

*Note.* ^1^Ratings of concentration disruption associated with intrusive memories was not completed if participants had zero intrusions; ^2^SEQ, Stress and Energy Questionnaire; ^3^SWEBO, Scale of Work Engagement and Burnout; ^4^ITL, Intention to leave job.

**Table S3.** All changes made to study materials and procedures based on participant feedback and the corresponding themes

| **Theme** | | **Participant feedback as expressed by one or more participants** | **Changes made to the study based on feedback** |
| --- | --- | --- | --- |
| **1** | Doing the digital intervention right | It is unclear what the next step is after filling out the baseline questionnaires, and after completing the assigned task. “*What happens now?”* (P1) | Added flowcharts that show the participant journey at the beginning and end of each module link that is sent out. It includes a green arrow with the text “You are here” to demonstrate where they are in the study, and the next step. |
| **2** | Getting the data right | It is difficult to remember exactly how many intrusions were had in the last week (retrospective rating taken at baseline). (P1) | We kept this question as part of inclusion criteria and ask for a general estimate (i.e. ‘have you had at least 2 intrusive memories in the last week’), but also added a baseline (week -1) daily electronic registration/diary of intrusive memories that participants are asked to complete during the week prior to filling in the baseline questionnaires and completing the intervention/control session. |
| **3** | Feeling that participation is right | It looks like there are going to be a lot of questions in the SmartTrial list of questionnaires. [You] could lump some of the single-item, or shorter questionnaires together. (P3) | We did this with 4 questionnaires, where we combined two of them together twice. We also removed one of the work-related questionnaires. |
| **4** | Feeling that participation is right | In the Demographics form where it asks about healthcare occupation, it should include “ambulance staff” as an option. (P1 & P3) | We had added “ambulance” to the list of options for this question, as well as in the list of examples of healthcare professions. |
| **5** | Getting the data right | [You] should be mindful of those who are on holiday when asking how many hours they have worked in the last month. (P1) | Added a question at the end of each intrusive memory diary that asks “How many day shift have you worked in the past week?” |
| **6** | Feeling that study participation is right | In the “Clinical Background” form, [you] should rephrase the question about asking if they have any illnesses to 'untreated/difficult to treat physical illnesses' (otherwise anyone over the age of 50 will write things here, e.g. ‘high blood pressure') (P1) | Changed the working to this question to say, “Do you have any current untreated/difficult to treat physical?”, instead of, “Do you have any physical illnesses?” |
| **7** | Getting the data right | Edit the help text for the LEC-5. Maybe add a help text? Need to make it clear that you can select several options (P1) | Bolded the instruction text, where it says “For each experience, mark one or several” of the options, to make it stand out more. |
| **8** | Getting the data right | Regarding the Demographics questionnaire, where it asks, “Do you have any specialisation?” this needs to be clarified as it is unclear how to interpret it. Maybe say “further education” instead. (P2 & P3) | Changed the phrasing from “specialisation” to “further education” as this is the correct phrasing in Swedish. |
| **9** | Getting the data right | It is difficult to answer the question about how these intrusions have impacted functioning, because the question refers to so many different aspects in life (job, school, etc.) – yet only one answer is supposed to capture all these things. This question could be broken down to ask more specifically how intrusions have impacted functioning in these different areas of life. (P3) | We broke this down into two questions: 1) “How much to your intrusive memories impact your functioning at work?” and 2) “How much do your intrusive memories impact your functioning in other areas of your life, for example: relationship with others, parenting, socially, studies, household work, volunteer work, etc?” We also included and optional free text response after each of these questions asking participant to briefly describe how so. |
| **10** | Getting the data right | Regarding the question about social support, this question will have a low rating by most, because healthcare professionals cannot talk about their work at home, even for difficult events (due to confidentiality) so they cannot really get support (i.e., relatives do not know about what they have been through.) [You] should add “colleagues” in the example or explanation of the question. (P3) | Added “colleagues” to the example in the question, so that it reads as, “How much support have you received from your colleagues, family, friends, or other after the traumatic event(s)?” |
| **11** | Getting the data right | Regarding the scope of work question in the Demographics questionnaire, it is too hard to calculate the number hours I have worked in the past month. It would be easier to answer if the question asked what percentage they have worked instead. (P1 & P3) | Changed this question in the baseline questionnaires to say, “What percentage do you work?” |
| **12** | Doing the intervention right | You should clarify before the video starts explaining “List of your intrusive memories” that the list will be made within the platform – so participants won't think they need to go get paper before the video starts to start writing them down. (P2) | Added an instruction in this part of the Intervention instructions, “NOTE! You do not need to write anything down now, but after you have watched the video.” |
| **13** | Doing the intervention right | The instructions for accessing Tetris from your smartphone/computer were a bit unclear, it was difficult to see where to turn off the 'Ghost Piece' function. You should make this clearer. (P2 & P3) | Edited the screenshot of where to turn off the ‘Ghost Piece’ function, by adding bolder rings around this in on the picture to make it stand out more, and by adding arrows that point to it. |
| **14** | Doing the intervention right | There are some ambiguities regarding tetris.com in terms of the advertising. You should let participants know that this will happen, that they can expect ads to appear when trying to access the game. (P2 & P3) | Added to the instruction page on how to access the game that there will be advertisements that appear, but they can be skipped. |
| **15** | Doing the intervention right | The tetris.com instructions for Tetris may be a bit confusing? Maybe they can be summarised into simpler bullet points. (P3) | Simplified the special gameplay instructions so that they are written and displayed as 3 steps, (e.g., 1. Remember to have the memory active…, 2. Play using ‘mental rotation’…etc.) |
| **16** | Doing the intervention right | It is unclear why the “Ghost Piece” function should be turned off. (P2) | Added to the Tetris gameplay instructions that this is an important aspect of the intervention for it work. Rather than a full theoretical explanation, to avoid participant burden. |
|  | Doing the intervention right | You should add that the video will open in new tab. (P1) | Added to the pages in the platform where a video appears for the first time, “The video will open in a new window. Please watch the video and them come back to this SmartTrial page.” |
| **17** | Getting the data right | Regarding the examples of COVID-related trauma events, instead of just having the last option be “other”, put it this way: “If none of the above examples are suitable for the traumatic event(s) you have experienced, you can give a brief description here:” (P1) | Added this to the example COVID-related events checklist. If participants answer “other” in the list of examples, they are then prompted to briefly describe their COVID-related trauma event if none of the above examples related to an experience they had. |
| **18** | Doing the intervention right | The step-by-step instructions for how to fill in the digital diary and how to play Tetris are too much. (P1 & P3) | We have slimmed down these instructions in the platform, and removed the screenshots that showed how to open the daily diary links, so that the instructions appear slimmer and simpler. |

**References**

Fýlkesnes, K., & Førde, O. H. (1992). Determinants and dimensions involved in self-evaluation of health. *Soc Sci Med, 35*(3), 271-279. doi:10.1016/0277-9536(92)90023-j

Holman, E. A., Silver, R. C., Mogle, J. A., & Scott, S. B. (2016). Adversity, time, and well-being: A longitudinal analysis of time perspective in adulthood. *Psychol Aging, 31*(6), 640-651. doi:10.1037/pag0000115

Hultell, D., & Gustavsson, J. P. (2010). A psychometric evaluation of the Scale of Work Engagement and Burnout (SWEBO). *Work, 37*(3), 261-274. doi:10.3233/wor-2010-1078

Kjellberg, A., & Wadman, C. (2002). Subjective stress and its relation to psychosocial work conditions and health complaints. A test of the Stress-Energy model. *Stockholm*.

Newby, J. M., & Moulds, M. L. (2010). Negative intrusive memories in depression: the role of maladaptive appraisals and safety behaviours. *J Affect Disord, 126*(1-2), 147-154. doi:10.1016/j.jad.2010.03.012

Rudman, A., Omne-Pontén, M., Wallin, L., & Gustavsson, P. J. (2010). Monitoring the newly qualified nurses in Sweden: the Longitudinal Analysis of Nursing Education (LANE) study. *Hum Resour Health, 8*, 10. doi:10.1186/1478-4491-8-10
